# Pre-operative hypoalbuminemia is associated with complication rate and overall survival in patients with vulvar cancer undergoing surgery

**DOI:** 10.1007/s00404-019-05278-7

**Published:** 2019-08-29

**Authors:** Christine Bekos, Stephan Polterauer, Veronika Seebacher, Thomas Bartl, Elmar Joura, Alexander Reinthaller, Alina Sturdza, Reinhard Horvat, Richard Schwameis, Christoph Grimm

**Affiliations:** 1 Department of Gynaecology and Obstetrics, Gynecologic Cancer Unit, Comprehensive Cancer Center, Medical University of Vienna, Waehringer Guertel 18-20, 1090 Vienna, Austria; 2 Karl Landsteiner Institute for General Gynecology and Experimental Gynecologic Oncology, Vienna, Austria; 3 Department of Radiation Oncology, Medical University of Vienna, Vienna, Austria; 4 Department of Pathology, Medical University of Vienna, Vienna, Austria

**Keywords:** Hypoalbuminemia, Albumin, Vulvar cancer, Prognosis, Complication

## Abstract

**Objective:**

Hypoalbuminemia, a known marker for malnutrition, has been associated with an increased risk for perioperative morbidity and poor prognosis in patients with solid tumors. The aim of this study was to investigate the prognostic and predictive value of pre-treatment serum albumin levels for survival and postoperative complications in patients with vulvar cancer undergoing surgery.

**Methods:**

Within in this retrospective study, we assessed data of 103 consecutive patients with vulvar cancer undergoing primary surgery into this study. Pre-treatment serum albumin levels were correlated with clinico-pathological parameters and complications. We performed univariate log-rank test and multivariable Cox regression models to evaluate the association between pre-treatment serum albumin and survival.

**Results:**

We found hypoalbuminemia (< 35 mg/dl) in 9 of 103 (8.7%) patients. No difference in tumor characteristics was observed between patients with hypoalbuminemia and normal serum albumin levels. Difference in postoperative complications (55.6% and 37.8% of patients with hypoalbuminemia and normal serum albumin levels, respectively) was not statistically significant (*p* = 0.345). Shorter overall survival (OS) was observed in patients with hypoalbuminemia (5-year OS rate 17.1%) when compared to patients with normal serum albumin levels (5-year OS rate 58.6%, *p* = 0.004). In multivariable analysis, age (*p* = 0.017), FIGO stage (*p* = 0.011) and serum albumin levels (*p* = 0.013) were independently associated with OS.

**Conclusion:**

Pre-treatment hypoalbuminemia is an independent prognostic biomarker for OS in patients with vulvar cancer. We did not find an association between pre-treatment hypoalbuminemia and a higher risk for postoperative complications.

**Electronic supplementary material:**

The online version of this article (10.1007/s00404-019-05278-7) contains supplementary material, which is available to authorized users.

## Introduction

Vulvar cancer is a rare disease, but the incidence has increased over the last 30 years [1]. Although this malignancy is mostly diagnosed in elderly patients, mean age at diagnosis is decreasing steadily [2]. Various prognostic parameters have been evaluated in vulvar cancer, but none of these has entered clinical practice to date [3–5].

Albumin is one of the most important proteins maintaining the colloid osmotic pressure, scavenging free radicals, providing ligand binding and drug transport, effecting vascular permeability and participating in intracellular pathways [6–8]. A reduced serum albumin concentration is associated with poor outcome in sepsis, heart failure, renal disease and cancer [9–11]. Various causes of hypoalbuminemia in patients with cancer have been described. The most important cause is an increased catabolism and following cachexia [12]. Furthermore, increased vascular permeability contributes to a shift of albumin from the intravascular sector towards the interstitium, leading to decreased serum albumin levels [13]. Comorbidities associated with cancer, such as sepsis, chronic liver or renal disease and gastrointestinal bleeding can also contribute to hypoalbuminemia by various mechanisms [14]. Hypoalbuminemia has been found to be of prognostic value in patients with endometrial [15], ovarian [16] and colorectal cancer [17]. In vulvar cancer patients, hypoalbuminemia has been shown to be associated with major postoperative wound complications [18].

The aim of this study was to investigate the association between pre-treatment serum albumin and postoperative complications and prognosis in patients with vulvar cancer undergoing surgery.

## Materials and methods

### Patients

309 consecutive patients diagnosed with and treated for vulvar cancer between 1996 and 2016 at the Comprehensive Cancer Center Vienna were initially identified. 175 had to be excluded due to missing laboratory results. 31 patients had to be excluded because they did not receive surgical therapy. Finally, 103 patients were enrolled in the present study. Data were retrospectively extracted from paper-based and electronic medical records. Prior to treatment, physical examination by a specialist in internal medicine was conducted and blood tests including serum albumin levels were performed as part of clinical routine in all patients. Patients with signs of inflammation or chronic liver disease were excluded from the study. Signs of inflammation were accessed searching for increased CRP concentrations, increased leucocyte counts or clinical signs of inflammation such as fever. The diagnosis of vulvar cancer was established by punch biopsy prior to surgery. Uniform criteria for surgical procedure terminology, pathologic variables, and sites of recurrence were used. Disease staging was based on the FIGO 2009 classification system [19].

Depending on the size of the lesion and the treating physician’s choice, patients underwent radical local excision, modified radical hemivulvectomy, modified radical posterior vulvectomy, modified radical anterior vulvectomy or radical vulvectomy. Groin lymph node dissection was performed if cancer invasion depth was > 1 mm. Ipsilateral lymph node dissection was performed for lateral lesions without clinically suspicious groins. Bilateral lymph node dissection was performed for midline lesions or when positive lymph nodes were suspected/found. Patients with smaller tumors (< 4 cm) were candidates for sentinel lymph node dissection using dual-staining with technetium 99 and blue dye application for detection. Active vacuum drainage with a diameter of 10–14 mm was used for groin surgery depending on the extent of lymphadenectomy. When the drainage fluid was lower than approximately 50 ml, the vacuum was removed and 1 day afterwards the drainage was removed.

Patients were instructed to clean local wounds by showering with warm water at least twice a day, resorbable sutures were used and were not removed. In selected cases local metronidazol was used for wound cleaning. In selected cases with high risk for wound infection prophylactic antibiotic was used.

Patient’s instruction was based on recent findings showing decreased symptom prevalence in women with counselling using evidence-based counseling guideline [20].

In cases of lymph node involvement, postoperative radiotherapy was applied according to standardized treatment protocols.

Postoperative complications were graded according to the Clavien–Dindo classification [21].

Patients’ continuous follow up was until hospital discharge. Afterwards follow-up was carried out every 3 months for the first year, every 6 months until the fifth year and annually up to 10 years. Complications risen after hospital discharge were assessed in our outpatient clinic. Follow up visits included vulvoscopy, vagino-rectal and groin palpation, and evaluation of serum squamous cell carcinoma antigen (SCC).

If any clinically suspicious lesion and/or tumor marker elevation was detected, biopsy and/or computed tomography was performed. Following standard clinical guidelines, recurrent disease was either diagnosed by biopsy or suspected by imaging methods.

The study was approved by the Ethics Committee of the Medical University of Vienna (IRB approval number: 1901/2017) before the study was initiated. Since this study was a retrospective analysis the ethics committee waived the requirement to obtain distinct informed consent from patients. The database with patients’ records was anonymized and de-identified prior to analysis.

### Albumin measurement

Blood samples (citrated plasma) for evaluation of serum albumin levels were taken by peripheral venous puncture within routinely performed blood tests prior to treatment. Serum albumin was assayed with bromocresol green using routine clinical chemical photometric analyzers [22]. By our institution’s laboratory, the normal range for serum albumin levels is defined between 35 and 52 mg/dl.

### Statistical analysis

Values are presented as mean values with standard deviation (SD) or total numbers or percentages (%). To compare mean serum albumin levels with clinico-pathological parameters Students’ *T* tests and one-way ANOVA tests were performed. *p* values of < 0.05 were considered statistically significant. To evaluate the independent risk factors for complication (CDC 1–5) binary logistic regression was performed, using all well-established parameters. To rule out a potential bias regarding prognostic value of hypoalbuminemia on OS patients, who died within 30 days after surgery, were excluded from this survival analysis. With respect to overall survival, differences between groups were tested using the log-rank test and presented as Kaplan–Meier survival curves. Multivariable analysis was performed using a Cox regression model including as independent variables serum albumin levels (dichotomized at 35.0 mg/dl), patients’ age (dichotomized at the median value of 68.2 years), tumor stage (FIGO III and IV vs. FIGO II vs. FIGO I), tumor grade (G3 vs. G2 vs. G1) and histology (squamous cell carcinoma vs. others) as independent variables. Statistical analyses were performed using the statistical software SPSS 24.0 for MAC (SPSS 24.0, IBM Inc., Armonk, NY).

## Results

Patients’ demographics are shown in Table 1.

**Table 1:** Patients' characteristics in 103 patients with vulvar cancer

Parameter	*N* (%) or mean (SD)
Total number of patients enrolled	103
Age at diagnosis (years)	69.04 (13.97)
ECOG status	
0	40 (38.8%)
1	26 (25.2%)
2	5 (4.9%)
3	4 (3.9%)
Unknown	28 (27.2%)
BMI	28.34 (5.10)
Histological type	
Squamous cell carcinoma	97 (94.2%)
Others	6 (5.8%)
Histological grade	
G1	24 (23.3%)
G2	63 (61.2%)
G3	16 (15.5%)
Tumor stage	
FIGO IA	17 (16.5%)
FIGO IB	45 (43.7%)
FIGO II	10 (9.7%)
FIGO IIIA	13 (12.6%)
FIGO IIIB	9 (8.7%)
FIGO IIIC	5 (4.9%)
FIGO IVA	4 (3.9%)
Treatment—surgery	
Radical local excision	35 (34.0%)
Modified radical hemivulvectomy	18 (17.5%)
Modified radical posterior vulvectomy	10 (9.7%)
Modified radical anterior vulvectomy	31 (30.1%)
Radical vulvectomy	9 (8.7%)
Lymphadenectomy—surgery	
No lymphadenectomy	20 (19.4%)
Unilateral lymph node dissection	24 (23.3%)
Bilateral lymph node dissection	59 (57.3%)
Systematic lymph node dissection (per groin)	66 (46.5%)
Sentinel lymph node dissection (per groin)	76 (53.5%)
Lymph node involvement	
Negative or not evaluated	75 (72.8%)
Positive	28 (27.2%)
Recurrence status	
No. of patients with recurrent disease	37 (35.9%)
Type of recurrence	
Local	28 (27.2%)
Distant	8 (7.7%)
Mean time to recurrent disease (months)	35.43 (34.80)
Status at last observation	
Alive with no evidence of disease or stable disease	56 (54.4%)
Progressive disease	5 (4.9%)
Tumor related death	22 (21.4%)
Dead as a result of other causes	20 (19.4%)
Mean time of follow-up (months)	44.08 (37.10)

*SD* standard deviation, *FIGO* International Federation of Gynaecology and Obstetrics, *CDC* Clavien–Dindo Classification

Mean age of patients was 69.04 and was therefore used as cut-off.

In the present study mean (SD) pre-treatment serum albumin was 41.34 (5.57) mg/dl. 9 (8.7%) patients were found to have hypoalbuminemia. No difference in tumor characteristics was observed between patients with hypoalbuminemia and normal serum albumin levels.

Mean albumin values broken down by clinico-pathological parameters are provided in Table 2.

**Table 2:** Relationship between clinico-pathological parameters and serum albumin in 103 patients with vulvar cancer

	Albumin ≤ 35 mg/dl	Albumin > 35 mg/dl	*p *value^1^
Tumor stage			0.577
FIGO I	4 (44.4%)	58 (61.7%)	
FIGO II	1 (11.1%)	9 (9.6%)	
FIGO III and IV	4 (44.4%)	27 (28.7%)	
Lymph node involvement			0.664
Negative or not evaluated	6 (66.7%)	69 (73.4%)	
Positive	3 (33.3%)	25 (26.6%)	
Age at first diagnosis (years)			0.214
≤ 69.04	2 (22.2%)	41 (43.6%)	
> 69.04	7 (77.8%)	56 (56.4%)	
ECOG			0.324
Unknown	2 (22.2%)	26 (28.7%)	
0–1	5 (55.6%)	61 (64.9%)	
2–3	2 (22.2%)	7 (7.4%)	
Histological grade			0.158
G1	3 (33.3%)	21 (22.3%)	
G2	3 (33.3%)	60 (63.8%)	
G3	3 (33.3%)	13 (13.8%)	
Histological type			0.479
Squamous cell carcinoma	8 (88.9%)	89 (94.7%)	
Others	1 (11.1%)	5 (5.3%)	

*FIGO* International Federation of Gynecologists and Obstetricians, *ECOG* Eastern Cooperative Oncology Group) performance status

^1^Chi-square test

Postoperative complications (mainly mild complications) were observed in 55.6% and 39.4% of patients with hypoalbuminemia and normal albumin levels, respectively (*p* = 0.345). Severe complications (CDC3-5) were rare and observed in 0% and 4.3% of patients with hypoalbuminemia and with normal albumin serum levels, respectively. Any kind of postoperative complication was observed in 42/104 patients. Mean albumin values broken down by Clavien–Dindo-classification are provided in Table 3.

**Table 3:** Numbers of postoperative complications classified according to Clavien–Dindo-Classification (CDC) broken down by pre-operative serum albumin levels (103 patients)

Perioperative complications (CDC)	Albumin ≤ 35 mg/dl	Albumin > 35 mg/dl	Complete cohort
None	4 (44.4%)	57 (60.6%)	61 (59.2%)
Grade 1	2 (22.2%)	17 (18.1%)	19 (18.4%)
Grade 2	3 (33.3%)	16 (17.0%)	19 (18.4%)
Grade 3a	0 (0%)	1 (1.1%)	1 (1.0%)
Grade 3b	0 (0%)	2 (2.1%)	2 (1.9%)
Grade 4	0 (0%)	0 (0%)	0 (0%)
Grade 5	0 (0%)	1 (1.1%)	1 (1.0%)
Total	9	94	103

27/103 (26.2%) patients received postoperative radiotherapy. Comparing nodal positive patients receiving adjuvant radiotherapy to those patients without postoperative radiation we could not find any significant difference in complication rates (10/27 vs. 32/76, *p* = 0.820).

Lymphadenectomy related complication rate such as lymphedema or lymph cysts according to no lymphadenectomy, sentinel lymphadenectomy and systemic lymphadenectomy was 0/20 (0%), 4/45 (8.9%) and 5/37 (13.5%), *p* = 0.215. Significantly more complications were observed when any kind of lymphadenectomy was performed. Mainly low risk complications were observed.

Complication rate according to type of operation was 12/35 (34.3%) in radical local excision, 6/18 (33.3%) in modified radical hemivulvectomy, 4/10 (40%) in modified radical posterior vulvectomy, 8/31 (25.8%) in modified radical anterior vulvectomy and 3/9 (33.3%) in radical vulvectomy, *p* = 0.311.

The American Society of Anesthesiologists (ASA) classification was available in 77 patients. The complication rate among patients with ASA 1 was 7/9 (77.8%), in patients with ASA 2 14/39 (35.9%), in patients with ASA 3 9/22 (40.9%) and 3/7 (42.9%) in patients with ASA 4, *p* = 0.15.

Most frequent types of complications were local wound infection (*n* = 20), partially requiring antibiotics (CDC 1–2), hemorrhage or hematoma with (*n* = 8), and lymphedema or lymphocele with (*n* = 9). One patient died 1 day after surgery due to myocardial infarction and concomitant pulmonary embolism. She was an 80-year-old woman with a FIGO stage II [pT2 N0 M0 R0] disease.

Table 4 provides results of multivariable logistic regression analysis including all patients of the study population showing the independent association of age (≤ 69.04 years vs. > 69.04 years), serum albumin levels (> 35 mg/dl vs. ≤ 35 mg/dl), ECOG status (0 vs. > 0) and Lymphadenectomy (no vs. yes) on probability for complications (CDC 1–5).

**Table 4:** Multivariate regression analysis of prognostic factors for complications (CDC 1–5), *N* = 102

Variable	CDC 1–5		
	OR	95% CI	*p* value
Age (years)			
≤ 69.04	1		
> 69.04	0.75	0.27–2.04	0.563
Albumin (mg/dl)			
> 35	1		
≤ 35	0.24	0.04–1.45	0.119
ECOG-status			
0	1		
> 0	0.65	0.24–1.73	0.387
Lymphadenectomy			
No	1		
Yes	4.22	1.04–17.05	0.043

*FIGO* International Federation of Gynecologists and Obstetricians, *ECOG* (Eastern Cooperative Oncology Group) performance status

In vulvar cancer patients with hypoalbuminemia compared to patients with normal serum albumin concentrations, 5-year overall survival (OS) rates were 17.1% and 58.6%, respectively (*p* < 0.004). In univariate survival analyses hypoalbuminemia (*p* < 0.004), advanced FIGO stage (*p* = 0.003) and patients’ age (*p* = 0.003) are associated with poor OS. In multivariable analysis, serum albumin (*p* = 0.013), FIGO stage (*p* = 0.011) and patients’ age (*p* = 0.017) are associated with poor OS. Results of the univariate Kaplan–Meier analysis and the multivariable cox regression model are shown in Table 5.

**Table 5:** Univariate and multivariate overall survival analyses in 103 patients with vulvar cancer

	Overall survival			
	Univariate^a^		Multivariate^b^	
	*p* value	5 year OS rate	*p* value	HR (95% CI)
Serum albumin (≤ 35 mg/dl vs. > 35 mg/dl)	0.004	17.1% vs. 58.6%	0.023	0.3 (0.1–0.8)
FIGO stage (I vs. II vs. III–IV)	0.003	63.8% vs. 64.3% vs. 34.3%	0.009	1.6 (1.1–2.3)
Patients’ age (< 69.04 vs. ≥ 69.04 years)	0.003	79.2% vs. 36.6%	0.015	2.4 (1.2–4.8)
Histological grade (G1 vs. G2 vs. G3)	0.574	68.2% vs. 54.0% vs. 45.5%	0.642	1.1 (0.7–2.0)
Type of surgery (local vs. radical)	0.463	54.3% vs. 56.5%	0.491	0.8 (0.4–1.5)
Type of Histology (squamous cell vs. others)	0.753	55.1% vs. 50.0%	0.129	0.3 (0.1–1.5)

*HR* hazard ratio, *95% CI* 95% confidence interval, *OS* overall survival

^a^Log rank test

^b^Multivariate Cox-regression analysis

Patient’s characteristics of the nine patients with hypoalbuminemia are displayed in Table 6.

**Table 6:** Patients' characteristics in nine vulvar cancer patients with hypoalbuminemia

PAT ID	Age	BMI	ASA	FIGO	N	OP	LNE	RT	Complication rate
1	80	n.a	3	III	Pos	Radical local excision	Systematic bilateral	Yes	CDC 1
2	33	n.a	1	III	Pos	Radical local excision	Systematic bilateral	Yes	CDC 1
3	86	18	4	I	Neg	Radical local excision	SLN unilateral	No	CDC 2
4	79	31.4	4	II	Neg	Modified radical ant vulvectomy	SLN unilateral	No	CDC 2
5	70	25.4	2	III	Pos	Radical local excision	Systematic bilateral	Yes	CDC2
6	80	26.5	2	I	Neg	Radical local excision	No	No	No
7	79	n.a	2	I	Neg	Modified radical anterior vulvectomy	Systematic bilateral	No	No
8	65	n.a	n.a	III	Neg	Radical local excision	No	Yes	No
9	83	n.a	n.a	I	Neg	Modified radical ant vulvectomy	SLN bilateral	No	No

*BMI* body mass index, *ASA* American Society of Anesthesiologists, *N* nodal status, *LNE* lymphadenectomy, *RT* radiotherapy, *n.a.* not available, *SLN* sentinel lymph node

**Fig. 1:**
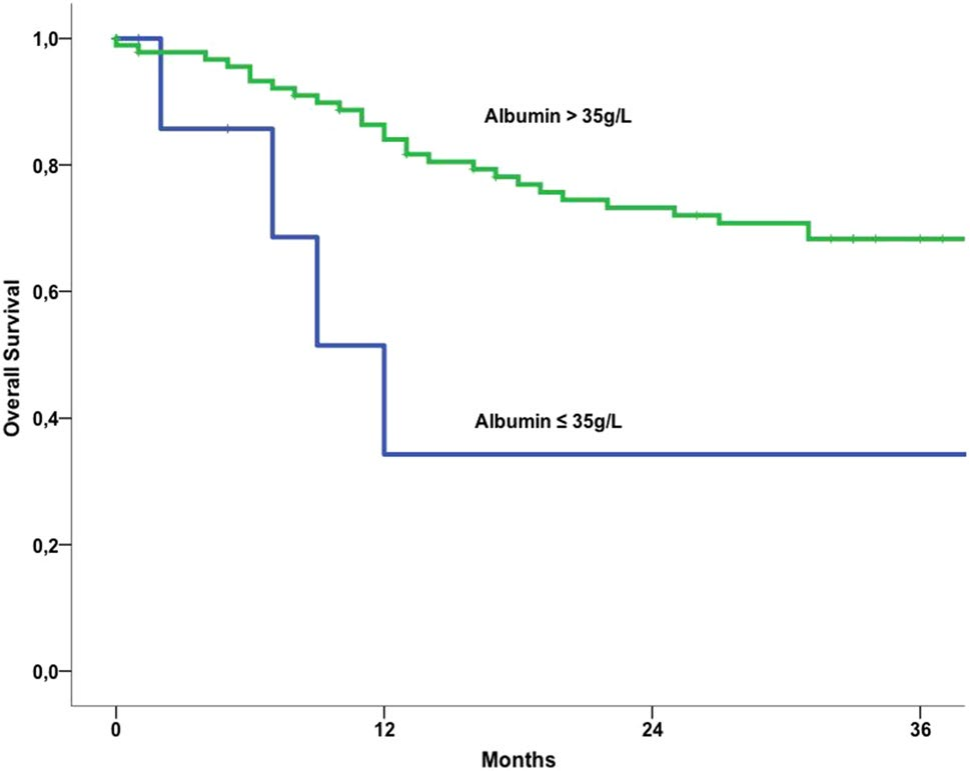
Kaplan–Meier overall survival curves in patients with vulvar cancer distributed by pre-therapeutic serum albumin levels

In Fig. 1 Kaplan–Meier survival curves demonstrate the association between pre-treatment serum albumin concentrations and overall OS.

## Discussion

Within the present study, we studied a relatively large consecutive series of patients with vulvar cancer and could demonstrate an association between pre-treatment hypoalbuminemia and poor prognosis. This is the first study investigating the prognostic role of pre-treatment hypoalbuminemia in patients with vulvar cancer.

In accordance with previously published literature in other solid tumors [15–17], our results suggest a strong association between low pre-treatment serum albumin levels and shorter OS in patients with vulvar cancer independent of other established prognostic parameters. These findings are biologically plausible, as hypoalbuminemia seems to be caused by increased catabolism subsequently leading to cachexia and additionally by advanced tumor stage [12]. Serum albumin is commonly used for assessing patients’ nutritional status. In advanced tumor stages, the levels of serum albumin can drop sharply, because both malnutrition and systematic inflammatory response due to tumors suppress albumin synthesis [23]. Malnutrition and cachexia in cancer patients are significant problems due to a variety of mechanisms involving the tumor, the host response to the tumor, and anticancer therapies [24]. Malnutrition has been associated with worsening of quality of life, reduced treatment response, increased risk of chemotherapy-induced toxicity and a reduction in OS [25]. Therefore, pre-treatment albumin may in future be a helpful tool to recognize a broader high-risk population and supply optimized therapy. The association between hypoalbuminemia and poor prognosis provides an opportunity to explore the role of pre-treatment nutritional interventions as well as increased follow up care.

Mild postoperative complications (CDC < 3) are common events in the surgical treatment of vulvar cancer. Despite a trend towards higher rate of complications in the group of patients with hypoalbuminemia, the observed difference in complications was not statistically significant. In a previously published report hypoalbuminemia was shown to be associated with major postoperative wound complications in women undergoing surgery for vulvar cancer [18]. In this study population, hypoalbuminemia was found in 12.8% of patients with vulvar cancer, which is comparable to the rate of hypoalbuminemia found in the current cohort (8.9%). Interestingly, the rate of complication was 10.4% compared to 40.3% in our cohort. A possible explanation for differences in complication rates between the two studies is that different complication classification systems were used. In the current analysis, any kind of complication was assessed and characterized by the Clavien–Dindo classification [21], while Sullivan et al. [18] registered only major wound complications defined as deep surgical site infection. In our cohort, we found major wound complications in 13.7% (*n* = 14), in 22.2% and 12.9% of patients with hypoalbuminemia and normal albumin levels, respectively (*p* = 0.438). Still, underreporting of complications is a common problem in retrospective studies, but these mistakes might be distributed equally between groups with normal albumin concentrations and hypoalbuminemia. The association between hypoalbuminemia and increased risk for postoperative complications is supported by a study including 2110 patients with gynecologic malignancies where patients with hypoalbuminemia were six times more likely to suffer from severe complications and were at ten-fold higher risk to die within 30 days after surgery [26]. In addition, these findings are in line with findings in patients with other gynecologic malignancies such as ovarian cancer [27].

In Gaarenstroom et al. [28], 76% of the patients suffered from postoperative complications. In contrast, the complication rate of 40.8% in our cohort is much lower. A possible explanation for this diverging results could be the much larger surgical extent in the above mentioned study where patients received modified radical vulvectomy and complete inguinofemoral lymphadenectomy. In our patient cohort 50% of patients receiving lymphadenectomy only underwent sentinel node dissection, which could be a possible explanation.

Hypoalbuminemia often reflects the presence of advanced disease and low performance status caused by tumor cachexia [12]. In our study cohort, presence of hypoalbuminemia was not associated with clinic-pathological parameters such as FIGO stage, patients’ age, and performance status. In contrast, in ovarian cancer hypoalbuminemia was associated with poor performance status and advanced FIGO stage [27]. In endometrial cancer hypoalbuminemia was inversely correlated with FIGO stage, histological grade, and patients' age [15]. These controversial findings might be caused by the small number of patients with hypoalbuminemia within our cohort. Of note, we observed a trend towards more advanced stage, older age and poor performance status in our cohort (Table 2).

The strengths of the present study include the single institution uniform approach to care and completeness of clinical data. The main potential limitation of this study—as typical for retrospective studies—is lack of random assignment, patient selection, and incomplete data acquisition. In addition, the number of patients with hypoalbuminemia in our cohort is small, therefore limiting the statistical power of our analyses.

Potential strengths of serum albumin as prognostic biomarker are low costs and broad availability [29].

In conclusion, this study suggests that hypoalbuminemia might be a useful prognostic biomarker for overall survival in women with vulvar cancer that is low in cost and broadly available [29]. This and whether serum albumin is associated with perioperative outcomes needs to be further investigated in larger well-controlled trials.

## Electronic supplementary material

Below is the link to the electronic supplementary material.

Supplementary material 1 (PNG 56 kb)
