# Supplementary figures and images for: Pre-operative hypoalbuminemia is associated with complication rate and overall survival in patients with vulvar cancer undergoing surgery
Source: Arch Gynecol Obstet. 2019 Aug 29;300(4):1015–22. doi: 10.1007/s00404-019-05278-7 (PMC6759670; doi:10.1007/s00404-019-05278-7)

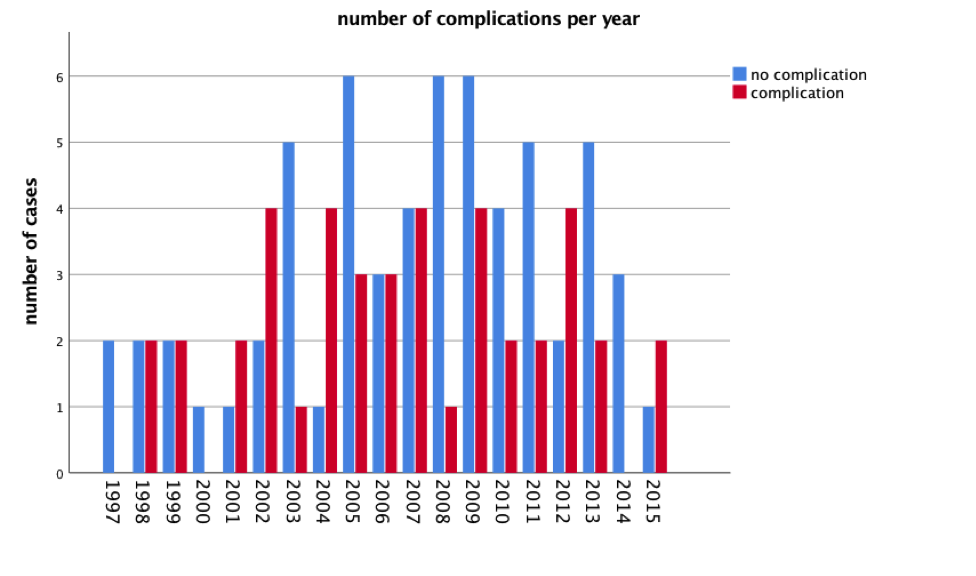

Supplement: Supplementary file 1 — Supplementary material 1 (PNG 56 kb) [file 404_2019_5278_MOESM1_ESM.png]
